# Auswirkungen vorgeschalteter Laborprozesse auf die Digitalisierung histologischer Schnittpräparate

**DOI:** 10.1007/s00292-024-01303-y

**Published:** 2024-02-22

**Authors:** Leander Schwaibold, Sven Mattern, Markus Mählmann, Leon Lobert, Thomas Breunig, Christian M. Schürch

**Affiliations:** Institut für Pathologie, Universitätsklinikum Tübingen, Liebermeisterstr. 8, 72076 Tübingen, Deutschland

**Keywords:** Mikrotomie, Kosteneinsparung, Digitaltechnologie, Gerätekontamination, Probenbearbeitung, Microtomy, Cost savings, Digital technology, Equipment contamination, Specimen handling

## Abstract

**Hintergrund:**

Viele Faktoren der Objektträger(OT)-Herstellung haben Einfluss auf Qualität und Datenmenge eines digitalisierten histologischen Schnittpräparates. Insbesondere die Reduktion von Verunreinigung sowie Auswahl des geeigneten Eindeckmaterials haben das Potenzial, Scanzeit und Datenmenge zu reduzieren.

**Ziel der Arbeit:**

Das Ziel dieser Arbeit ist die Objektivierung von Beobachtungen aus dem Prozess der Digitalisierung unseres Institutes, um den Einfluss von Laborprozessen auf die Qualität digitaler Histologiepräparate zu ermitteln.

**Material und Methoden:**

Versuch 1: Einscannen von OT vor und nach Installation einer Mittelkonsole im Mikrotomiebereich zur Reduktion von Schmutz und statistische Auswertung der erhobenen Parameter. Versuch 2: Erneutes eindecken von OT (nach Abschluss der Diagnostik) mit Glas und Folie. Einscannen der OT und statistische Auswertung der erhobenen Parameter.

**Schlussfolgerung:**

Die gezielte Umstrukturierung im Laborprozess führt zu einer Reduktion von OT-Kontaminationen. Dies bewirkt eine signifikante Reduktion der Datenmenge und Scanzeit von digitalisierten Schnitten. Folie als Eindeckmaterial verursacht im Gegensatz zu Glas weniger Prozessfehler im weiteren Verlauf. Nach unseren Schätzungen führt dies zu deutlichen Kosteneinsparungen.

## Abstract (en)

**Background:**

Several factors in glass slide (GS) preparation affect the quality and data volume of a digitized histological slide. In particular, reducing contamination and selecting the appropriate coverslip have the potential to significantly reduce scan time and data volume.

**Goals:**

To objectify observations from our institute’s digitization process to determine the impact of laboratory processes on the quality of digital histology slides.

**Materials and methods:**

Experiment 1: Scanning the GS before and after installation of a central console in the microtomy area to reduce dirt and statistical analysis of the determined parameters. Experiment 2: Re-coverslipping the GS (post diagnostics) with glass and film. Scanning the GS and statistical analysis of the collected parameters.

**Conclusion:**

The targeted restructuring in the laboratory process leads to a reduction of GS contamination. This causes a significant reduction in the amount of data generated and scanning time required for the digitized sections. Film as a coverslip material minimizes processing errors in contrast to glass. According to our estimation, all the above-mentioned points lead to considerable cost savings.

Im Mittelpunkt der digitalen Pathologie steht das Einscannen eines Objektträgers (OT) zur Generierung eines digitalen Schnittpräparates. Unterschiedliche Prozessschritte in der Herstellung eines OT haben signifikante Auswirkungen auf verschiedene Parameter der Digitalisierung. In dieser Arbeit werden Beobachtungen aus dem Prozess der Digitalisierung des eigenen Institutes durch 2 Versuchsreihen untersucht. Wir konnten zeigen, dass der Grad der Verunreinigung eines OT Einfluss auf die beim Einscannen erzeugte Datenmenge und Scanzeit hat. Auch wenn die Auswahl des Eindeckmediums keinen unmittelbaren Effekt auf den Speicherbedarf zeigt, gibt es doch Vor- und Nachteile, die im digitalen Prozess berücksichtigt werden sollten. Durch einfache Umstrukturierung von Laborprozessen konnte die Anzahl an Verunreinigungen vermindert und der Scanprozess optimiert werden.

Die moderne Pathologie erfährt zurzeit einen Wandel von der klassischen Lichtmikroskopie hin zur Befundung von digitalisierten histologischen Schnittpräparaten am Computer (Whole Slide Imaging, WSI).

Noch existiert keine eindeutige Definition des Begriffs „Digitale Pathologie“. Manche Autor:innen verstehen hierunter lediglich die Digitalisierung eines OT. Jedoch beinhaltet die vollständige Digitalisierung eines Institutes für Pathologie weitere tiefgreifende Schritte und Optimierungsprozesse in allen Arbeitsbereichen. Die European Society of Digital and Integrative Pathology (ESDIP) definiert Digitale Pathologie als sämtliche Technologien, welche dazu beitragen, Workflows zu optimieren [1].

Die Digitalisierung der Pathologie verspricht viele Vorteile, wie beispielsweise erleichterte Durchführbarkeit von Telepathologie. So kann durch das Teilen digitaler Schnittpräparate auf die Expertise von Kolleg:innen zugegriffen werden, ohne die Notwendigkeit, OT per Post an die jeweiligen Institute zu versenden. Auch besteht die Möglichkeit der Anbindung kleinerer Kliniken an spezialisierte pathologische Zentren durch Einrichtung eines Scanners vor Ort. Diese Umstellungen ermöglichen eine bessere und notwendige Verteilung der Arbeitslast. In Zukunft wird die Anzahl der zu befundenden Fälle pro Patholog:in zunehmen, was unter anderem auf die steigende Inzidenz von Krebserkrankungen bei abnehmender Anzahl praktizierender Patholog:innen zurückzuführen ist [2–4]. Die Arbeitsgruppe um Mikkelsen et al. erwartet beispielsweise eine jährliche Zunahme pathologischer Fälle in Süddänemark von 4,5 % [3]. Der Fachkräftemangel in der Pathologie wird von vielen Autor:innen als globales Problem angesehen, von dem auch Deutschland betroffen ist [5]. Die Anzahl von Einwohner:innen pro Patholog:in beträgt in Deutschland 48.000, was der zweitniedrigsten Rate in Europa entspricht. Es ist zu erwarten, dass auch in Deutschland die stetig steigende Arbeitsbelastung aufgrund einer zu geringen Anzahl an Berufseinsteiger:innen in der Pathologie in Zukunft nicht adäquat abgedeckt werden kann [2].

Die Digitalisierung eines Institutes für Pathologie ist ein zeitaufwendiger und kostspieliger Prozess. Da OT auch in einem vollständig digitalisierten Institut nach wie vor hergestellt werden müssen, ist das Scannen eines histologischen Schnittpräparates ein zusätzlicher Arbeitsschritt im Laborprozess [6]. Allerdings zeigen Erfahrungsberichte aus bereits digitalisierten Instituten, dass sich der finanzielle Mehraufwand im Laufe der Zeit ausgleicht und auf längere Sicht sogar Kosten eingespart werden. Die Arbeitsgruppe um Hanna et al. erwartet in ihrem Institut bspw. eine Kosteneinsparung von 1,3 Mio. $ in einem 5‑jährigen Zeitraum (2014–2018). Grund hierfür sei unter anderem eine Verringerung des Personalaufwands. Zusätzlich entfallen z. B. Transportkosten von Glas-OT innerhalb des Instituts als auch Kosten für physische Lagerung von Glas-OT [7].

Das Department für Pathologie und Neuropathologie des Universitätsklinikums Tübingen durchläuft derzeit eine grundlegende Umstellung sämtlicher Laborprozesse und Arbeitsabläufe auf digitale Workflows. Die Qualität des im Labor hergestellten Glas-OT korreliert mit der Qualität des WSI [8, 9]. Insbesondere wirkt sich die Art des Eindeckmaterials durch die ungleiche Verteilung von Luftblasen auf Qualität, Scanzeit und Datenmenge eines digitalisierten histologischen Schnittpräparates aus [8]. Ferner sieht der *Leitfaden Digitale Pathologie* des Bundesverbands Deutscher Pathologen e. V. vor, dass sämtliche sog. relevante Partikel auf dem OT vom Scanner erkannt und eingescannt werden müssen. Als relevante Partikel werden Zellverbände, die aus mindestens 3 × 3 = 9 Zellen bestehen, bzw. Areale mit einer Mindestbreite von 30 µm definiert [10]. Zahlreiche Verunreinigungen auf den OT erfüllen diese Kriterien und werden daher als Gewebe erkannt und ebenfalls gescannt, sog. Gewebeartefakte („tissue artefacts“) [11]. Unsere Hypothese war, dass nicht nur Luftblasen, sondern auch der Grad der Verunreinigung des eingescannten Glas-OT mit der erzeugten Datenmenge und Scanzeit eines WSI korreliert. Vor diesem Hintergrund wurden sämtliche an der OT-Herstellung beteiligten Prozesse in unserem Institut einer gründlichen Analyse und Optimierung unterzogen. Dies umfasst den Präparatzuschnitt und die Makroskopie, das Einbetten und Ausgießen der Blöcke, das Schneiden am Mikrotom sowie die Färbung und das Eindecken der OT. Ziel dieser Arbeit ist es, die im Umstellungsprozesse festgestellten Einflussfaktoren auf Scanzeit und Speicherplatzbedarf zu objektivieren und die Relevanz des Einflusses zu ermitteln. Nach unserem Wissen existiert bislang keine Studie, welche die Minimierung von OT-Verschmutzungen durch Umstrukturierung von Laborprozessen beschreibt, mit dem Hintergrund, die Digitalisierung der Pathologie zu optimieren.

## Materialien und Methoden

### Laborvoraussetzungen und durchgeführte Maßnahmen

**Figure 1:**
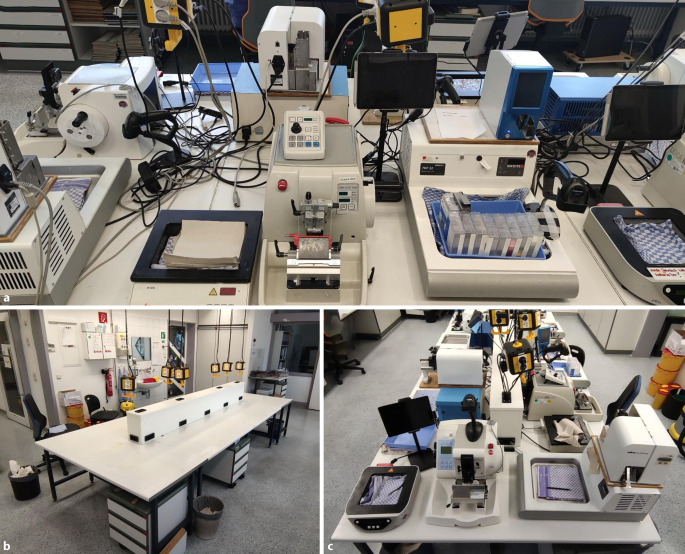

Das Institut verfügt über 3 Zuschnitt- und Färbelabore. Das Labor mit dem höchsten Arbeitsaufkommen hat einen zentralen Mikrotomiebereich, der Platz für 7 medizinisch-technische Fachkräfte (MTA) bietet. Im Rahmen der Optimierungsprozesse wurde dieser einer Grundreinigung und Umstrukturierung unterzogen. Jede Arbeitsstation ist mit einem beheizten Wasserbecken, Mikrotom, Tablet-PC und OT-Drucker ausgestattet. Die räumliche Nähe dieser Geräte ist für einen effizienten Ablauf essenziell. Ein Risiko ist jedoch die Kontamination frisch gedruckter OT mit im Prozess anfallenden Paraffinspänen des Mikrotoms durch das obligate Trimmen vor dem eigentlichen Schneidevorgang. Ferner bieten freiliegende Kabel Sammelflächen für Paraffinspäne, Staub und weitere Verunreinigungen. Wir implementierten eine Mittelkonsole (Abb. 1), welche alle freiliegenden Kabel bündelt und die Arbeitsflächen für eine regelmäßige Reinigung zugänglich macht.

Um die Verunreinigungen weiter zu reduzieren, wurde die Durchführung des täglichen Reinigungsprozesses der Mikrotomarbeitsplätze auf eine dedizierte Person übertragen. Zudem werden die OT-Drucker einmal wöchentlich durch die unterwiesene Person gereinigt und gewartet (Reinigung von Objektträgerkassetten, Führungen, Rollen und Druckköpfen, Kontrolle der Druckbänder). Das gesamte Labor wird im 3‑monatigen Turnus einer Grundreinigung unterzogen.

### Probenauswahl und Einverständnis

Um die Auswirkung der im Labor installierten Mittelkonsole auf die Scanqualität zu ermitteln, analysierten und verglichen wir den Grad der Verunreinigung an 120 Hämatoxylin-Eosin(HE)-gefärbten OT vor und nach der Implementierung der Mittelkonsole. Die OT stammen aus dem Archiv des Instituts für Pathologie und Neuropathologie. Für den Vergleich von Glas und Folie wurden von 20 archivierten Paraffinblöcken von Plazentagewebe nach Abschluss der Diagnostik erneut jeweils 2 OT von Schnitten in Serie (*n* = 40 OT) angefertigt, HE-gefärbt und je ein OT mittels Glas (Tissue-Tek Glas, Sakura Seiki Co. Ltd., Nagano, Japan; Microm Cover Tech CTM6, Microm International GmbH, Deutschland) und ein OT mittels Folie (CM1260, Pathotape, Histoserve GmbH, Celle, Deutschland; Tissue-Tek Film, Tissue-Tek Film Eindeckfolie, Sakura Seiki Co. Ltd., Nagano, Japan) eingedeckt. Das Vorliegen eines Einverständnisses zur Verwendung für wissenschaftliche Zwecke wurde überprüft.

### Ethik

Die Untersuchungen wurden im Rahmen der Umstellungsmaßnahmen der Labore und deren Optimierung durchgeführt. Alle Patientendaten wurden anonymisiert. Die Informationen einzelner Personen spielen im Rahmen der Untersuchung keine Rolle, sodass ein Ethikvotum nicht notwendig ist.

### Ermittlung der Datenbasis

Die ausgesuchten OT wurden retrospektiv mit dem Philips SG300 Pathology Scanner (Philips, Amsterdam, Niederlande)gescannt und im Image-Management-System (IMS) Philips IntelliSite Pathology Solution 5.1 (Philips, Amsterdam, Niederlande) strukturiert abgelegt und erfasst.

Um die Datenbasis zur statistischen Auswertung erstellen zu können, wurden die im iSyntax-Format gescannten OT mit folgenden Parametern exportiert: Whole Slide Image (WSI); Format: tiff; Vergrößerung: 40fach; Qualität: 100; Dateiformat: BigTIFF.

Die 240 exportierten OT (vorher und nachher) entsprachen einer zu analysierenden Datenmenge von 1,6 TB. Die 64 exportierten OT (Glas und Folie) entsprachen einer zu analysierenden Datenmenge von 426 GB. Zur Analyse wurde QuPath (Version 0.4.3, [12]) genutzt. Im ersten Schritt wurden folgende Differenzierungen vorgenommen: Scanfläche (SF; gesamte Scanfläche des WSI), vom Scanner erkannte Gewebefläche (SGF; alle als Gewebe erkannten Partikel, inklusive aller Verunreinigungen).

Zur Ermittlung dieser Flächen wurden 2 Pixelklassifizierungen angewendet, die den gesamten Scan heranziehen. Die SGF wurde im ersten Schritt erkannt (Parameter: Resolution: Very low (7,95 µm/px); Channel: Average channels; Prefilter: Gaussian; Smoothing: 2; Threshold: 230; Above threshold: Ignore; Below threshold: Region; Region: Everywhere).

Die Ermittlung der Gesamtfläche des Scans erfolgte im zweiten Schritt (Parameter: Resolution: Very low (7,95 µm/px); Channel: Average channels; Prefilter: Gaussian; Smoothing: 0.5; Threshold: 250; Above threshold: Ignore; Below threshold: Region; Region: Everywhere). Die Ergebnisse wurden in den Annotationen des jeweiligen OT abgelegt.

Über die Splitfunktion wurde das befundrelevante Gewebe von Verschmutzungen und Gewebefragmenten differenziert. Auf dieser Basis wurden Gewebeobjekte als „befundrelevant“ klassifiziert und entsprechend annotiert. Die befundrelevante Gewebefläche (BRG) wird fortan als „Befundfläche“ bezeichnet. Aus dieser Klassifizierung ergibt sich, dass alle weiteren Objekte als Verschmutzung und Gewebefragmente klassifiziert und annotiert wurden („nicht befundrelevant“). Alle Annotationen wurden im folgenden Schritt als objektträgerzugehörende Textdateien exportiert.

Alle exportierten Textdateien wurden mit einem Microsoft-Powershell-Script für die statistische Auswertung in Microsoft Excel (Version 16.78, Microsoft Corporation, Redmond, WA, USA) aufbereitet, sodass alle statistikrelevanten Informationen datentypenkorrekt bereitgestellt werden konnten.

Zur Absicherung und Ergänzung dieser zuvor gewonnen Daten wurden zu jedem OT die zugehörenden Metadaten (Start des Scans, Ende des Scans, Größe des Scans in Byte, Scanspalten, Scanzeilen) aus dem IMS ergänzt.

### Statistik

Statistische Signifikanzen wurden mittels t‑Test für unabhängige (Versuchsreihe Mittelkonsole) und abhängige (Versuchsreihe Eindeckmaterial) Stichproben unter Verwendung von GraphPad Prism (Version 10.0.3, GraphPad Software, Boston, MA, USA) ermittelt. Die Berechnung des Standardfehlers des Mittelwerts und die (nichtlineare) Regressionsanalyse erfolgten ebenfalls mit GraphPad Prism.

## Beobachtungen und Resultate

### Reduktion von Schmutz durch Implementierung einer Mittelkonsole

**Figure 2:**
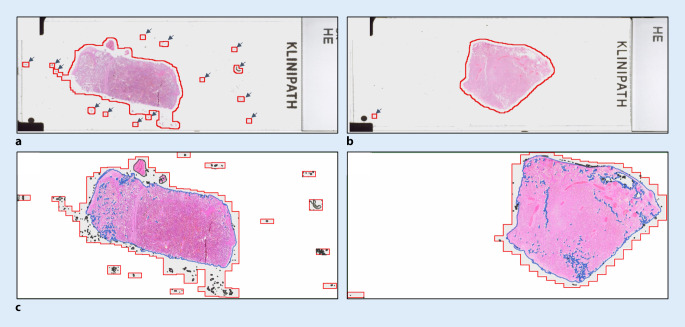

Die Installation einer Mittelkonsole hat zu einer Reduktion von Kontaminationen geführt. Dennoch lässt sich auch nach der Implementierung eine residuelle Kontamination der OT nachweisen (Abb. 2). Durch die Übertragung der Verantwortung auf eine einzelne Person wurde eine optimale Reinigungsstrategie für kritische Laborbereiche etabliert. Diese Veränderungen reduzierten die Kontaminationen.

### Schmutz verlängert die Scanzeit

**Figure 3:**
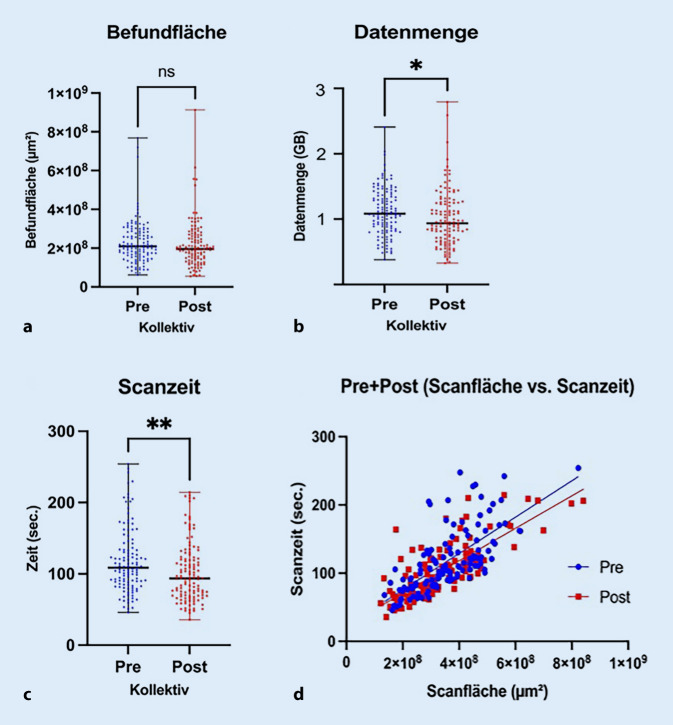

Die beiden Kollektive wiesen keinen Unterschied in der befundrelevanten Gewebefläche (BRG) auf. Auch die Scanfläche (SF) und die vom Scanner erkannte Gewebefläche (SGF) unterschieden sich nicht. Die Reduktion von Verunreinigungen bewirkte signifikante Veränderungen in 2 der betrachteten Parameter (Abb. 3b, c). Die durchschnittliche Datenmenge von 1,11 ± 0,03 GB/WSI erfuhr durch die Implementierung eine signifikante Reduktion auf 0,96 ± 0,04 GB/WSI. Die Scanzeit verringerte sich signifikant von 118,29 ± 4,18 s/WSI auf 101,84 ± 3,80 s/WSI. Während durchschnittlich eine ähnliche Datenmenge pro Sekunde gescannt wurde, konnten nach der Implementierung durchschnittlich 6,15 OT mehr pro Stunde gescannt werden (+19,03 %; Tab. 1). Der Vergleich einer nichtlinearen Regressionsanalyse der Scanzeit und Scanfläche zeigt, dass nach der Implementierung mehr Fläche in weniger Zeit gescannt wurden. Je größer die zu scannende Fläche war, desto deutlicher war dieser Unterschied (Abb. 3d).

**Table 1:** 

	Vorher	Nachher	*p*‑Wert
Anzahl OT (*n*)	120	120	–
Datenmenge (GB)	1,11 ± 0,03	0,96 ± 0,04	0,0402
Scanzeit (s)	118,29 ± 4,18	101,84 ± 3,80	0,0039
Datendurchsatz (MB/s)	9,99 ± 0,27	10,26 ± 0,25	n. s
Scanfläche (µm^2^)	3,61 × 10^8^ ±1,07 × 10^7^	3,32 × 10^8^ ±1,24 × 10^7^	n. s.
Vom Scanner erkannte Gewebefläche (µm^2^)	2,20 × 10^8^ ± 7,49 × 10^6^	2,14 × 10^8^ ± 9,09 × 10^6^	n. s.
Befundfläche (µm^2^)	2,28 × 10^8^ ±1,03 × 10^7^	2,18 × 10^8^ ±1,09 × 10^7^	n. s.
Verhältnis Gewebefläche/Scanfläche	0,61 ± 0,01	0,65 ± 0,01	0,0403
OT/h	32,32	38,47	–

Werte angegeben als Median +/− SEM (Standardfehler des Mittelwerts)

*OT* Objektträger, *n.* *s.* nicht signifikant

### Schmutz kann zu Geräteausfall und -stillstand führen

Verunreinigungen durch Paraffin- und Glasstaub o. Ä. können die Hardware von OT-Druckern und Computern/Tablets schädigen und somit zu einem Geräteausfall führen, z. B. durch reduzierte Abluft und damit Hitzeentwicklung oder durch Interferenzen mit Druckerköpfen oder beweglichen Teilen. Neben den Maßnahmen zur Schmutzreduktion wurde die Verantwortung für die Instandhaltung des Arbeitsbereiches zentralisiert. Die Reinigung erfolgte nach einem festen Plan.

Vor dieser Reorganisation kam es durchschnittlich zu 4 Ausfällen einzelner Komponenten an den Zuschnittarbeitsplätzen pro Woche. Die durchschnittliche Ausfallzeit betrug etwa 25 min und war betriebsverhindernd. Nach der Reorganisation kam es im Durchschnitt eines Monats zu einem Vorfall, der jedoch in etwa 10 min bereinigt werden konnte und lediglich betriebsbehindernd war.

### Einfluss des Eindeckmaterials auf Scanqualität und -zeit

Als Eindeckmaterial kommen 2 Materialien in Betracht: Glas und Folie. In einem Institut wie unserem, bei dem das Eindecken mit Glas der aktuelle Standard war, ist die Implementierung von Folie mit initial erhöhten (Anschaffungs‑)Kosten verbunden. Darüber hinaus ist Folie aufwendiger zu handhaben als Glas. Dennoch weist sie einige Vorteile für die Digitalisierung auf. Die Folie als Eindeckmaterial hat eine höhere Flexibilität gegenüber Glas, sodass eventuelle Unebenheiten des eingebetteten Gewebes (beispielsweise überstehender Knochen) besser abgedichtet werden. Dies minimiert das Risiko von Lufteinschlüssen und wirkt sich positiv auf Scanqualität und Datenmenge aus.

Folie erhöht auch die Stabilität der OT, wenn diese mechanisch belastet werden (Transport, Versendung, Handling). Hierbei entsteht im Gegensatz zu Glas kein Glasstaub durch etwaige Reibung der Materialien gegeneinander. Glasstaub kann vom Scanner versehentlich als Gewebe interpretiert werden und Scanzeit sowie Datenmenge erhöhen.

Im Laboralltag kann es dazu kommen, dass nach dem Eindecken Glas bzw. Folie nicht optimal mit dem Rand des OT abschließt. Fällt dies nicht frühzeitig in der Prozesskette auf, werden diese schlechter eingedeckten OT gescannt. Überstehende Eindeckgläser führen bei Entnahme oder Rückgabe des OT durch den Greifer des Scanners zu mechanischen Problemen. OT können während des Handlings im Scanner abbrechen und unbrauchbar werden. Fallen Überstände beim Eindecken mit Glas auf, so werden diese oft manuell abgebrochen. Dabei entsteht eine nicht unerhebliche Verletzungsgefahr und die Qualität des OT sinkt deutlich, da beim Abbrechen überstehenden Glases Lufteinschlüsse entstehen können und die nicht glatte Bruchkante ggf. bei der Gewebeerkennung des Scanners als zu scannendes Gewebe erkannt wird. Hierbei werden darüber hinaus oft deutliche Fingerabdrücke hinterlassen, die ebenfalls qualitätsmindernd sind.

Die durchschnittliche Datenmenge betrug bei mit Glas eingedeckten Proben 1,09 ± 0,07 GB/WSI und bei Folie 1,11 ± 0,07 GB/WSI. Somit wurde trotz der o. g. Beobachtungen keine signifikante Reduktion der Datenmenge beobachtet (*p* = 0,0986; Daten nicht im Detail gezeigt).

## Diskussion

Unsere Daten zeigen eine signifikant reduzierte Scanzeit und signifikant reduzierten Speicherplatzbedarf durch die Implementation einer Mittelkonsole und die damit verbundene Reduktion von Schmutzpartikeln im Mikrotomiebereich. Dabei stellt die Verschmutzung selbst keinen signifikant höheren Anteil an erkanntem Gewebe dar. Dies lässt darauf schließen, dass die Anordnung der Partikel eine relevante Rolle in den zugrundeliegenden Algorithmen für sowohl die mechanische Organisation des Scanprozesses als auch die spätere Speicherung haben. Insgesamt scheinen sich die detektierten Effekte weniger softwareseitig als vielmehr hardwareseitig niederzuschlagen.

Zudem zeigen die Daten, dass die Wahl des Eindeckmaterials in unserem untersuchten Kollektiv den Speicherplatzbedarf nicht signifikant beeinflusst. Die von Ferreira et al. erhobenen Ergebnisse bzgl. der vermehrten Datenmenge bei händisch mittels Glas im Vergleich zu maschinell mittels Folie eingedeckten OT konnten wir bei maschineller Eindeckung von sowohl Glas als auch Folie nicht bestätigen. Der mögliche Einfluss des händischen Eindeckens wurde bereits im Rahmen der genannten Arbeit diskutiert [8].

Folie hat als Eindeckmaterial gegenüber Glas Vorteile. Es entsteht kein Glasabrieb und keine Splitterung. Dies reduziert mechanische Probleme in den Geräten (z. B. im OT-Drucker oder im Eindeckautomat selbst). Auch reduziert sich die Verletzungsgefahr durch unsachgemäß eingedeckte OT. Folie ist zudem verformbar und führt bei der mechanischen Bearbeitung im Scanner zu weniger Prozessabbrüchen oder gar Glasbruch bei Verkantung der OT.

Zusammenfassend zeigen unsere Beobachtungen, dass sämtliche Prozessschritte einen wesentlichen Einfluss auf die Handhabung des OT selbst oder mindestens einen der Aspekte Scanqualität, Datenmenge oder Scandauer eines virtuellen OT haben. Jeder Teilprozess der OT-Herstellung ist somit qualitätsbestimmend.

Im Rahmen dieser Arbeit fokussierten wir uns auf größere Gewebestücke und explizit nicht auf Biopsien. Der Effekt bei Biopsien könnte aufgrund des insgesamt geringeren „tatsächlichen Gewebes“ noch größer sein als bei Zuschnittpräparaten. Auch implizieren die Ergebnisse, dass die Anordnung bei Biopsien eine entscheidende Rolle für die Effizienz des Scanprozesses haben könnte.

Alle in dieser Arbeit durchgeführten Untersuchungen erfolgten mit einem Scannermodell (Philips SG300), welcher zur Gewebedetektion die sog. Tile-Methode verwendet [13]. Hierbei werden das erkannte Gewebe sowie ein (je nach Modell individualisierbarer) Sicherheitsabstand um das Gewebe eingescannt. Jedoch unterscheiden sich die Detektionsmethoden zum Teil deutlich zwischen verschiedenen Scannermodellen unterschiedlicher Herstellern. Bei der Bounding-Box-Methode wird beispielsweise das erkannte Gewebe in einem quadratischen Areal begrenzt und anschließend die gesamte Fläche dieses Bereichs eingescannt, ohne zwischen eigentlichem Gewebe und leeren Arealen zu unterscheiden [13–15]. Wenn sich Verunreinigungen auf dem Objektträger befinden, könnte dies zu einem deutlich größeren Scanareal führen als bei der Tile-Methode. Dies wiederum würde die Scanzeit verlängern und in einer höheren Datenmenge pro WSI resultieren. Diese Hypothese wurde in der vorliegenden Arbeit allerdings nicht untersucht. Da die Vorgaben bzgl. Partikelgröße jedoch festgelegt sind [10], stellen Kontaminationen ungeachtet der genauen Heuristik des Scanners ein Problem dar. Vergleichende Analysen zwischen verschiedenen Modellen können hier in Zukunft helfen, allgemeingültige Empfehlungen auszusprechen.

Insgesamt sind weitere Untersuchungen auf diesem Gebiet erforderlich, um aus den sich ergebenden neuen Möglichkeiten für die Abläufe der einzelnen Institute und Praxen, aber auch im Sinne der Nachhaltigkeit die bestmöglichen Ergebnisse zu erzielen.

## Fazit für die Praxis

- Sauberkeit ist neben der Relevanz für die Patientensicherheit (Kontamination) auch ein relevanter Faktor betreffend Kosten und Zeit.
- Die Lokalisation von Partikeln auf dem Objektträger (OT) beeinflusst die Scangeschwindigkeit.
- Durch einfache Maßnahmen lassen sich Scanzeit und Speicherplatz von Whole Slide Images signifikant reduzieren.
- Dies reduziert die Kosten für Scannerbetrieb, Speicherplatz, Energieverbrauch und Personal.
- Die Minimierung von Kontaminationen auf OT ist somit ein nicht zu vernachlässigender Kosten- und Qualitätsfaktor und sollte als Querschnittsprozess mit eigenen Organisationsfaktoren innerhalb der gesamten Teilprozesse betrachtet werden.

## Abkürzungen

BRG: Befundrelevantes Gewebe
ESDIP: European Society of Digital and Integrative Pathology
GB: Gigabyte
HE: Hämatoxylin-Eosin
IMS: Image-Management-System
MB: Megabyte
MTA: Medizinisch-technische Fachkraft
OT: Objektträger
SF: Scanfläche
SGF: Vom Scanner erkannte Gewebefläche
TB: Terabyte
WSI: Whole Slide Image
